# Imported Infectious Disease and Purpose of Travel, Switzerland

**DOI:** 10.3201/eid1302.060847

**Published:** 2007-02

**Authors:** Lukas Fenner, Rainer Weber, Robert Steffen, Patricia Schlagenhauf

**Affiliations:** * University of Zürich, Zürich, Switzerland; 1 Current affiliation: University Hospital Basel, Basel, Switzerland

**Keywords:** visiting friends and relatives, travel, traveler, infectious disease, malaria, diarrhea, pre-travel advice, research

## Abstract

We evaluated the epidemiologic factors of patients seeking treatment for travel-associated illness from January 2004 through May 2005 at the University Hospital of Zurich. When comparing persons whose purpose of travel was visiting friends and relatives (VFR travelers; n = 121) with tourists and other travelers (n = 217), VFR travelers showed a distinct infectious disease and risk spectrum. VFR travelers were more likely to receive a diagnosis of malaria (adjusted odds ratio [OR] = 2.9, 95% confidence interval [CI] 1.2–7.3) or viral hepatitis (OR = 3.1, 95% CI 1.1–9) compared with other travelers but were less likely to seek pre-travel advice (20% vs. 67%, p = 0.0001). However, proportionate rates of acute diarrhea were lower in VFR (173 vs. 364 per 1,000 ill returnees). Travel to sub-Saharan Africa contributed most to malaria in VFR travelers. In countries with large migrant populations, improved public health strategies are needed to reach VFR travelers.

## Abstract 2

Travelers who visited friends or relatives were more likely to receive a diagnosis of malaria or viral hepatitis than those who traveled for other reasons.

More than 800 million tourist arrivals were registered worldwide in 2005, and an estimated 2% of the world’s population lives outside the country of birth (*1*). Importation of infectious diseases to new countries is likely to increase among both travelers and immigrants. Approximately 80 million people from resource-rich areas worldwide travel to resource-poor countries every year (*2*) and are exposed to many infections that are no longer prevalent in the countries where they live. Travelers visiting friends and relatives (VFR travelers)—predominantly immigrants and their children returning to their home countries for vacations, to maintain family ties, or to visit sick relatives—are at particularly high risk for preventable infectious diseases, such as malaria, typhoid fever, hepatitis A, hepatitis B, and tuberculosis (*3*–*5*).

A recent review of a global surveillance network’s data set showed different demographic characteristics and different types of travel-related illnesses among immigrant-VFR, traveler-VFR, and tourist travelers (*5*). The population of western Europe includes ≈20 million persons living in nonnative countries; most are settled immigrants. One third were born in a country outside of Europe (*6*). In Switzerland, ≈21% (1.6 million) residents are foreign born (*7*). Compared with the health of the native population of Switzerland, the health status of the immigrant population is poor (*8*) because of the high prevalence of infectious diseases in the home countries (*9*), a difficult psychosocial environment in the new country, inappropriate risk-taking behavior (*10*), and social inequalities (*11*).

The University Hospital of Zürich serves a large proportion of the city’s population, which includes a multiethnic range of patients and immigrants. The outpatient departments treat ≈120,000 patients each year, and the inpatient departments treat >35,000. We evaluated the epidemiology of imported infectious disease of patients seeking treatment for travel-associated illness at the University Hospital of Zürich from January 2004 through May 2005.

## Patients and Methods

The University Hospital of Zürich, as part of the global GeoSentinel surveillance network, contributed clinician-based surveillance data during a 17-month period, January 2004–June 2005, according to demographic characteristics, risk for infectious disease while traveling, and frequency of pretravel advice. GeoSentinel is a global sentinel surveillance network that was established in 1995 through the International Society for Travel Medicine and the US Centers for Disease Control and Prevention. The network consists of 33 globally distributed member travel/tropical medicine clinics (*12*) and has been widely used to document travel-related illnesses (*5*,*13*–*15*).

### Inclusion Criteria

To be eligible, patients must have crossed an international border ≤10 years before seeking treatment and must have sought medical advice for a presumed travel-related illness. Relevant travel details focused only on data from the 6 months before the onset of illness. Only final diagnoses were considered, and >1 diagnosis per patient was possible. Data were collected according to a standardized, anonymous questionnaire. The questionnaire asked for demographic data (age, sex, country of birth, country of residence, current citizenship), travel history during the previous 5 years, inpatient or outpatient status, major clinical symptoms (>1 per patient possible), pretravel visit information, reason for most recent travel, and patient classification. Reasons for most recent travel were immigration, tourism, business, research/education, missionary/volunteer work, visit to friends or relatives, and expatriation. Patients were classified as immigrants/refugees, foreign visitors, urban expatriates, nonurban expatriates, students, military personnel, or travelers. Working and final diagnoses were assigned by a physician.

### Definitions

An immigrant/refugee was defined as a foreign-born person who had obtained permanent resident status or immigrant/refugee status in Switzerland. Traveler (or traditional traveler) was defined as a resident of Switzerland who crossed an international border and did not previously immigrate to Switzerland. When the purpose of recent travel was visiting friends and relatives, a traveler was termed VFR. Different patient classifications were possible (i.e., immigrant-VFR, traveler-VFR). The rate of illness was calculated as the number of patients with a specific or a summary diagnosis as a proportion of all VFR or traditional travelers, respectively, expressed as number per 1,000 patients. The percentage of “chief complaints” was expressed as the number of primary symptoms that led to a clinic visit per total patients in each group. More than 1 chief complaint per patient was possible.

Countries were assigned to 1 of 15 regional classifications (*13*). Because of small case numbers, a more simplified regional classification was sometimes used: sub-Saharan Africa, south-central America (South and Central America), Asia (south-central, southeast, east, and north Asia), and eastern Europe. “All other regions” include those with no assigned travel destination. For travelers or VFR who entered >1 region, the most likely place of exposure during travel was determined to be the single region visited.

Summary diagnosis were defined as follows: “respiratory tract infection” included upper and lower respiratory infections; “malaria” infections included all malaria-causing species; “diarrhea” included acute diarrhea of parasitic, viral, bacterial or unknown origin; “hepatitis” included chronic or acute viral hepatitis; “viral syndrome” included any nonspecific viral symptoms; and “AIDS/HIV/STI” included asymptomatic HIV, acute HIV, AIDS, gonorrhea, syphilis, and other sexually transmitted infections (STIs). Syndrome groups such as “dermatologic disorder” were defined as previously described (*15*).

### Statistics

Stata software (version 9.1, Stata Corporation, College Station, TX, USA) was used for statistical analysis. Odds ratios (OR) of binary, categorical, or continuous variables were determined by logistic regression (multivariate or univariate) and adjusted to age and sex if indicated. Statistical significance of dichotomous variables was achieved by using χ² or nonparametric tests.

## Results

### General Description and Demographic Data

We analyzed 451 patients included in the database: 181 immigrants, 227 travelers, 25 foreign visitors, and 18 others (expatriates, students, military personnel). Age range was 16–87 years (median 33, interquartile range 27–43); 48% were female, and 20% were inpatients. The median duration of travel was 17.5 days (interquartile range 13–29 days). For these patients, 671 diagnoses were counted. Leading complaints were “fever” (43.0%), “gastrointestinal” (42.7%), “head-ear-nose” (25.2%), “respiratory” (24.3%), “musculoskeletal” (12.8%), and “skin” (11.9%, data not shown). The visits were evenly distributed during the calendar year, with no seasonal abnormities or significant associations.

### Comparison of VFR and Traditional Travelers

Our analysis included 217 traditional travelers and 121 VFR travelers. For traditional travelers, the reason for most recent travel was tourism or business. Most VFR travelers (86%) were in the category “immigrants.” Birth country regions of VFR travelers were Asia (30%), sub-Saharan Africa (24%), Eastern Europe (17%), and Central or South America (11%). The basic demographic pattern was comparable (Table 1). VFR travelers traveled on average for a longer period than traditional travelers, were slightly older, were more likely to have inpatient status, and were less likely to seek pretravel advice. Traveled regions were also comparable (Table 2). Fever and gastrointestinal disorders were the most frequent reasons for seeking treatment (Table 2). Traditional travelers had more gastrointestinal symptoms (53.91% vs. 39.66%, p = 0.03). When the disease spectrums were compared, acute diarrhea was more often diagnosed in traditional travelers (26%) than in VFR travelers (11%). The summary diagnosis HIV/AIDS/STI was more commonly established in VFR travelers (9.9% vs. 4.3%); the same was true for malaria (7.7% vs. 2.7%). The proportionate illness patterns are shown graphically in the Appendix Figure.

**Table 1:** Demographics data on persons included in the study whose purpose of travel was visiting friends and relatives (VFR) versus traditional travelers (travelers)

	Travelers, no. (%) n = 217	VFR, no. (%) n = 121	p value
Sex			
Male	119 (54.8)	61 (50.4)	0.43
Female	98 (45.2)	60 (49.6)	
Age (y)			
Median	32	39	0.008
Interquartile range	32–46	26–45	
Patient type			
Outpatient	185 (84.5)	84 (70.6)	0.002
Inpatient	34 (15.5)	35 (29.4)	
Travel duration (d)			
Median	15	21	0.006
Interquartile range	11–24	14–31	
Sought pretravel advice?			
Yes	65 (67)	18 (20)	0.0001
No	32 (33)	70 (80)	
Traveled region			
Sub-Saharan Africa	43 (19.81)	27 (22.31)	
Asia	61 (28.11)	21 (17.35)	
Eastern Europe	6 (2.76)	21 (17.35)	
Central/South America	22 (10.13)	9 (7.43)	
All other regions	85 (39.17)	43 (35.53)	

**Table 2:** Primary symptoms of persons seeking treatment at a clinic, frequent summary diagnosis, and syndrome groups in persons whose purpose of travel was visiting friends and relatives (VFR) versus traditional travelers (travelers)

	Travelers, no. (%)*	VFR, no. (%)*
Primary symptom		
Fever	108 (49.76)	57 (47.10)
Gastrointestinal	117 (53.91)	48 (39.66)
Head-ear-nose	54 (24.88)	38 (31.40)
Respiratory	52 (23.96)	34 (28.09)
Musculoskeletal	25 (11.52)	22 (18.18)
Skin	30 (13.82)	14 (11.57)
Fatigue	24 (11.05)	13 (10.74)
Other	18 (8.29)	16 (13.22)
Total	428	242
Summary diagnosis and syndrome groups		
Diarrhea, acute	79 (26.33)	21 (11.53)
Respiratory infection	40 (13.33)	22 (12.09)
HIV/AIDS	12 (4)	15 (8.24)
Malaria, all species	8 (2.67)	14 (7.69)
Viral syndrome	23 (7.67)	10 (5.49)
Viral hepatitis, acute/chronic	6 (2)	10 (5.49)
Urinary tract infection	3 (1)	3 (1.65)
Febrile illness, unspecified	10 (3.33)	1 (0.55)
Dengue fever (uncomplicated)	4 (1.33)	1 (0.55)
Sexually transmitted infection	1 (0.33)	3 (1.65)
Loa loa	–	2 (1.1)
Cutaneous leishmaniasis	1 (0.33)	–
Typhoid/paratyphoid fever	1 (0.33)	1 (0.55)
Brucellosis	–	1 (0.55)
Extraintestinal amebiasis	1 (0.33)	–-
Dermatologic disorder	22 (7.33)	9 (4.95)
Chronic diarrhea	7 (2.33)	5 (2.75)
Healthy	4 (1.33)	2 (1.1)
Adverse drug or vaccine reaction	3 (1)	1 (0.55)
Cardiovascular disorder	2 (0.67)	3 (1.65)
Neurologic disorder	2 (0.67)	2 (1.1)
Lost to follow-up	2 (0.67)	–
Pulmonary embolism	1 (0.33)	2 (1.1)
Psychological disorder	1 (0.33)	2 (1.1)
Death	1 (0.33)	1 (0.55)
Other diagnosis	66 (22)	51 (28.02)
Total	300	182

*Percentage expressed as number of primary symptoms that led to a clinic visit per total patients in each group.

When comparing VFR with traditional travelers, VFR travelers were more likely to receive a diagnosis of malaria, acute or chronic viral hepatitis, and HIV/AIDS/STI (Table 3) but less likely to receive a diagnosis of acute diarrhea. In contrast, traditional travelers were more likely to receive a diagnosis of diarrhea (OR 2.1, 95% confidence interval [CI] 1.2–3.6, p = 0.007; data not shown). Respiratory diseases and viral syndromes were significantly associated with VFR travelers only in the univariate analysis (Table 3). Traditional travelers were significantly more likely to seek pretravel advice compared with VFR travelers (Table 1).

**Table 3:** Association of infectious disease in persons whose purpose of travel was visiting friends and relatives (VFR) versus traditional travelers (travelers)*.

	Odds ratio	p value	95% CI
Univariate analysis			
HIV/AIDS/STI	2.42	0.019	1.15–5.07
Malaria	3.04	0.014	1.25–7.40
Diarrhea, acute	0.36	0.0001	0.21–0.61
Viral hepatitis	2.84	0.046	1.01–7.97
Respiratory infection	0.89	0.692	0.51–1.55
Viral syndrome	0.7	0.362	0.32–1.50
Multivariate analysis (adjusted to age and sex)			
HIV/AIDS/STI	2.63	0.014	1.21–5.69
Malaria	2.93	0.021	1.17–7.32
Diarrhea, acute	0.47	0.007	0.27–0.81
Viral hepatitis	3.15	0.032	1.10–9.02

*CI, confidence interval; STI, sexually transmitted infection.

A different infectious disease spectrum and a trend toward a distinct pattern in both VFR and traditional travelers were also found when selecting different travel regions (Figure). Malaria cases were almost exclusively imported from the sub-Saharan Africa region; 33.3% of diagnoses after travel to this region were attributed to malaria in VFR travelers, compared with 12.3% in traditional travelers. In total, 27 malaria cases were recorded in the GeoSentinel database during the 17-month period: 14 in VFR travelers, 8 in tourist travelers, 4 in recent immigrants, and 1 in an immigrant/refugee. Of these, 22 cases were imported from sub-Saharan Africa and 1 from Turkey; for 4 case-patients, no specified travel region or no information on place of exposure was available. When data were stratified by VFR versus traditional traveler, the risk for malaria in sub-Saharan Africa was twice as high in the VFR traveler group than in the traditional traveler group (data not shown).

**Figure:**
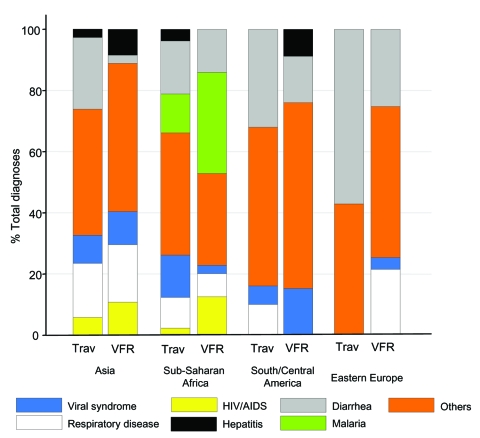
Percentage of disease diagnoses in travelers visiting friends and relatives (VFR) and traditional travelers (trav) who reported illnesses after returning to Switzerland, classified by geographic region visited.

## Discussion

The GeoSentinel site based at the University Hospital of Zürich represents a large population in Switzerland. However, GeoSentinel is a health facility–based surveillance system and does not actively screen for certain diseases. Patients included in the database do not necessarily represent the whole population or the epidemiology or frequency of the disease. Besides the unknown number of ill returned travelers going to general practitioners or nonspecialized clinics, the number of travelers returning in good health is also unknown. Incidence rates or relative risks therefore cannot be estimated. Similarly, patients with mild or self-limiting disease are likely to see a general practitioner rather than to go to a specialized center, although many VFR travelers do not have a regular general practitioner. On the other hand, Zürich is a large city with a socioculturally mixed population that offers an opportunity to study immigrant-VFR travelers, and many of these patients may prefer to go to a more anonymous university hospital than to a general practitioner. A limitation of the study is the relatively small number of patients included in the database during the 17-month period, which made it necessary to form summary diagnoses and regions.

In our analysis, VFR travelers showed a different infectious disease and risk spectrum than did traditional travelers; were more likely to receive a diagnosis of malaria, viral hepatitis, or HIV/AIDS/STI; and were less likely to seek pretravel advice. Traditional travelers (mainly tourists) were significantly more likely to seek advice before traveling and to have a posttravel diagnosis of acute diarrhea. This is consistent with previous studies from European migrants returning to their home countries (*16*), as well as a recent review of the global GeoSentinel database (*5*). Malaria is most likely to be acquired in the sub-Saharan Africa region, according to our data and those of others (*13*,*15*).

By contrast, acute diarrhea was the greatest problem in traditional travelers, with an illness rate of 364 per 1,000 ill returned travelers compared with 173/1,000 in VFR travelers. Acute diarrhea, or traveler’s diarrhea, is known to affect >50% of travelers, depending on the destination (*17*). The protective effect in VFR travelers could reflect immunity due to recent exposure or exposure in childhood.

Acute or chronic viral hepatitis was also significantly associated with VFR travel, which correlates with a recent study of hepatitis A virus infections in Swiss travelers during a period of 12 years that identified VFR travelers as a high-risk group, especially children of immigrants (*18*). Other significant associations of disease between VFR and traditional travelers were not found; however, this does not necessarily mean that no such relationship exists.

Systemic febrile illnesses, including malaria and typhoid fever, tuberculosis, and respiratory syndromes, are more frequently diagnosed among VFR travelers (*5*). In our study, respiratory diseases contributed to the relatively high rate of illness in both VFR and traditional travelers (181 vs. 184 per 1,000 ill returnees). No significant association could be established between influenza, long trip duration, and travel involving visiting friends and relatives as described before (*14*), probably because of small numbers and very few cases of influenza. Viral syndrome, a rather loosely defined summary diagnosis with unspecific viral symptoms, was also frequently diagnosed and can be interpreted as a flulike syndrome. Other typical tropical infectious diseases, such as typhoid fever, leishmaniasis, dengue fever, or brucellosis, were rarely diagnosed.

This study shows that VFR travelers are at greater risk for certain infectious diseases and have a disease spectrum distinct from that of traditional travelers. Malaria is the most important, life-threatening imported disease for both nonimmune and VFR travelers, and malaria acquisition is even more likely in VFR travelers. For other infectious diseases, HIV and STIs must also be included in the differential diagnosis, particularly for VFR travelers. VFR travelers are vulnerable because they may visit more rural destinations, live under poor sanitary conditions, and stay away for longer periods (*3*,*4*). Moreover, the health condition of the immigrant population in Switzerland is poor compared with that of the native population (*8*). Prevalence gaps in disease and disparities in access to care exist not only between countries but also between population groups within countries.

In addition, VFR travelers often did not seek pretravel advice. Thus, culturally sensitive strategies for pretravel contact with VFR travelers are greatly needed. Further surveillance of traveler groups with denominator data is needed, and prospective studies focusing on behavioral aspects of disease prevention would allow for evidence-based interventions as part of a public health strategy.

## Supplementary Material

Appendix Figure Illness rates in persons whose purpose of travel was visiting friends and relatives (VFR) versus traditional travelers (travelers). Points indicate number of illnesses per 1,000 ill returned travelers.
