# Supplementary figures and images for: Imported Infectious Disease and Purpose of Travel, Switzerland
Source: Emerg Infect Dis. 2007 Feb;13(2):217–22. doi: 10.3201/eid1302.060847 (PMC2725840; doi:10.3201/eid1302.060847)

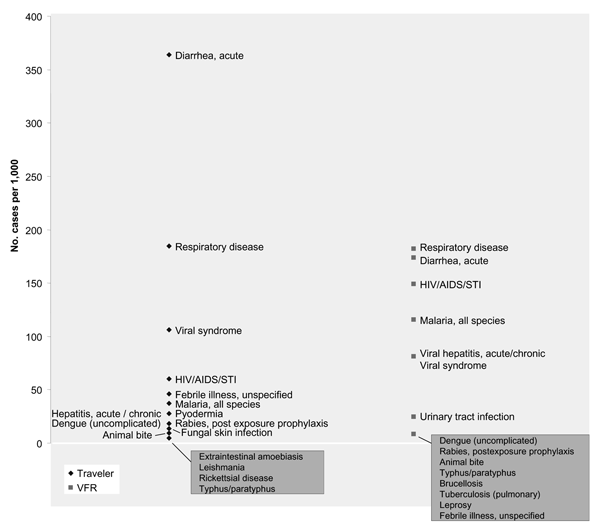

Supplement: Appendix Figure — Illness rates in persons whose purpose of travel was visiting friends and relatives (VFR) versus traditional travelers (travelers). Points indicate number of illnesses per 1,000 ill returned travelers. [file 06-0847_app-s1.gif]
